# Current and Future Distribution of the Tropical Tree *Cedrela odorata* L. in Mexico under Climate Change Scenarios Using MaxLike

**DOI:** 10.1371/journal.pone.0164178

**Published:** 2016-10-12

**Authors:** Israel Estrada-Contreras, Miguel Equihua, Javier Laborde, Enrique Martínez Meyer, Lázaro R. Sánchez-Velásquez

**Affiliations:** 1 Ambiente y Sustentabilidad, Instituto de Ecología A.C., Xalapa-Enríquez, Veracruz, Mexico; 2 Ecología Funcional, Instituto de Ecología A.C., Xalapa-Enríquez, Veracruz, Mexico; 3 Departamento de Zoología, Instituto de Biología, Universidad Nacional Autónoma de México, Mexico City, Mexico; 4 Instituto de Biotecnología y Ecología Aplicada, Universidad Veracruzana, Xalapa-Enríquez, Veracruz, Mexico; Pacific Northwest National Laboratory, UNITED STATES

## Abstract

Climate change is recognized as an important threat to global biodiversity because it increases the risk of extinction of many species on the planet. Mexico is a megadiverse country and native tree species such as red cedar (*Cedrela odorata*) can be used to maintain forests while helping mitigate climate change, because it is considered a fast growing pioneer species with great economic potential in the forestry industry. In order to assess possible shifts in areas suitable for *C*. *odorata* plantations in Mexico with ecological niche models, we used the MaxLike algorithm, climate variables, the geo-referenced records of this species, three general circulation models and three scenarios of future emissions. Results show a current potential distribution of 573,079 km^2^ with an average probability of occurrence of 0.93 (± 0.13). The potential distribution area could increase up to 650,356 km^2^ by 2060 according to the general circulation model HADCM3 B2, with an average probability of occurrence of 0.86 (± 0.14). Finally, we delimited an area of 35,377 km^2^ that has a high potential for the establishment of *C*. *odorata* plantations, by selecting those sites with optimal conditions for its growth that are outside protected areas and are currently devoid of trees. *C*. *odorata* has a significant potential to help in the mitigation of the effects of climate change. Using MaxLike we identified extense areas in Mexico suitable to increase carbon sequestration through plantations of this highly valued native tree species.

## Introduction

There is substantial consensus recognizing that the climate on Earth is changing and that human activities are the main cause of the increase in the concentration of greenhouse gases in the atmosphere, which is the main factor driving the change [1]. There are reliable estimates that from 1880 to 2012, the air temperature close to the surface of Earth’s landmasses and that of the oceans increased by about 0.85°C. It is estimated to be likely that by 2100 the temperature will increase by 2.6 to 4.8°C [2]. The effect of this general upward trend on the global water cycle will not be uniform. Precipitation contrasts between wet and dry areas and between seasons will increase, which in turn will have a great and varied influence on forest structure and dynamics around the world [2, 3]. A large fraction of both terrestrial and freshwater species will face increased extinction risk under projected scenarios of climate change during and beyond the 21st century, particularly as climate change interacts with other stressors, such as habitat modification, overexploitation, pollution, and the arrival of invasive species [3, 4, 5, 6].

Increased tree mortality and associated forest dieback is projected to occur in many regions over the 21st century, due to increased temperatures and drought [6]. Forests have two important roles with respect to global warming: the retention of carbon in the standing forest and the regulation of carbon flow by absorption through photosynthesis and through emission from clearing, the latter of which can be reduced by avoiding deforestation and counteracted by restoring woodlands [7]. Carbon is stored in the leaves, branches, trunks, and roots of trees as well as in forest soils. Old-growth tropical forests store 120 to 400 tonnes/ha of carbon [8]. Even without the loss of forest cover it is estimated that the increase in soil temperature from global warming has the potential to release massive amounts of carbon into the atmosphere, forming an additional positive feedback for climate change [9].

Human intervention in forests to reduce the sources (emission) or enhance the sinks of greenhouse gases is an interesting approach to mitigation that we have to balance with the current acceleration in biodiversity loss, which in turn threatens the provisioning of many ecosystem services to humans [10]. Several forest-related options are available for climate change mitigation: maintaining or increasing forest area, reducing deforestation, increasing afforestation (mainly by establishing plantations) and reforestation, increasing the use of wood products from sustainably managed forests and increasing long-term carbon storage in timber products [11, 12]. The most cost-effective mitigation options in forestry seems to be afforestation, sustainable forest management, and reducing deforestation, with large differences in their relative importance across regions [10]. Reforestation and afforestation can be very effective at sequestering carbon and can make great contributions to rural livelihoods and biodiversity conservation [13]. Ideally, plantations should provide environmental and socio-economic benefits so as to reduce pressure on natural forests, offset fossil fuel consumption, help to meet biomass demand, and provide rural employment [11].

Several projections made by different authors suggest a likely future scenario is one where the climate in Mexico will be warmer, rainfall lower and seasonality will shift. In addition, the hydrological cycle will become more intense, increasing the number of severe storms and the intensity of drought periods [14]. Mexico is one of the few mega-diverse countries on the planet. In spite of having more than 23,000 species of vascular plants [15], few forest species are of industrial interest in the country. Red cedar (*Cedrela odorata* L.; nomenclature follows Tropicos.org [16]), is the second most important tropical timber species in the forest industry in Mexico [17], only surpassed by mahogany (*Swietenia macrophylla*). It has great economic potential for its excellent features and high commercial timber value [18, 19]. *C*. *odorata* is found in the humid and semi-dry tropical areas of Mexico. It grows in tropical rain forest and tropical semi-deciduous forest in the lowlands; however, it also grows in the foothills of mountain ranges at the lower limits of the cloud forest, pine forest and pine-oak forest [20, 21].

There are commercial plantations of *C*. *odorata* in different regions of the country, for which growth rates of up to 2 m in height per year have been recorded [22, 23, 24]. In addition to its importance in the timber industry, it is widely used in traditional medicine, to produce honey, handicrafts and ornaments, erosion control and for the conservation of soil fertility. In some regions, it is planted as a hedge and windbreak to protect pastures and farmland [23]. Recently *C*. *odorata* was listed in the Official Mexican Standard NOM-059-SEMARNAT-2010 in the category “Subject to special protection” (Pr), which has caused some decrease in the establishment of red cedar plantations supported by the National Forestry Commission (CONAFOR, the institution responsible for forestry in Mexico). Since it is now subject to special protection, trade in wood from plantations or even timber from a few trees (often grown by farmers on their land), has become complicated, discouraging the management and utilization of the species.

Nevertheless, *C*. *odorata* still is a species of notable economic and social importance and its potential as an environmental aid that can help to mitigate the effects of climate change is highly relevant, given its rapid growth and ability to colonize sites devoid of woody vegetation. The aim of this paper is to assess the geographical areas that potentially meet the conditions most conducive to the presence of *C*. *odorata* in Mexico under climate change scenarios.

## Methods

### Study area

**Fig 1:**
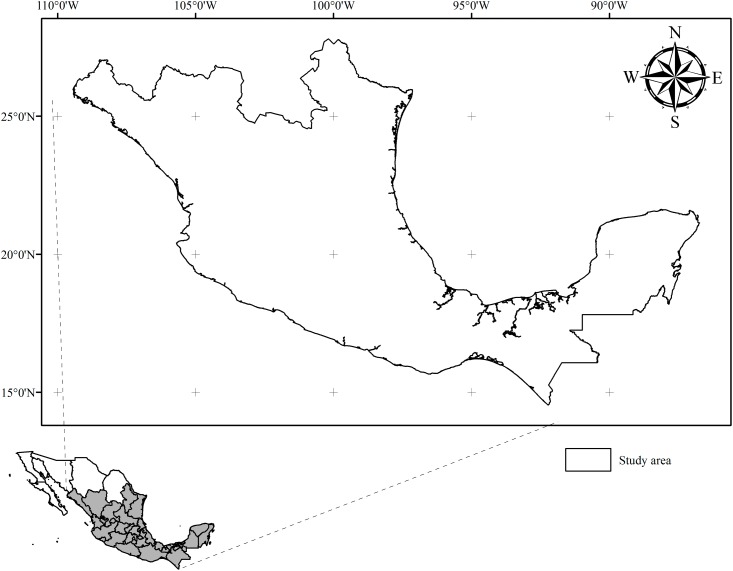
Study area for the potential distribution of *C*. *odorata* in Mexico.

Red cedar is a tree belonging to Family Meliaceae that can reach heights of 20–35 m. Native to tropical America, it can be found wild from Mexico (latitude 26° N) to northern Argentina (latitude 28° S). [21, 23]. In Mexico, *C*. *odorata* occurs along the coast of the Gulf of Mexico, from southern Tamaulipas and southeast of San Luis Potosí to the Yucatan Peninsula, and on the Pacific coast from Sinaloa to Guerrero and in the Central Depression and on the coast of Chiapas [21]. Our study area extends from 27°42'2.33'' to 14°32'9.13'' N latitude, and from 109°26'40.85'' to 86°44'51.94'' W longitude (Fig 1), covering a total area of 1,318,613 km^2^ (nearly 67% of the country), which corresponds in our database to 1,533,271 pixels with a resolution of 0.00833333 decimal degrees (~ 0.86 km^2^) each.

### Maps of the potential distribution of *Cedrela odorata*

Ecological niche modeling is a method that makes use of environmental variables, combining them with occurrence records for the species of interest to model its ecological requirements and predict its potential geographic distribution [25, 26]. A variety of statistical methods are available for estimating occurrence probabilities from presence-only, presence-absence, or presence-background data [27, 28], but by far the most widely used has been MaxEnt software, a presence-background machine-learning algorithm based on the principles of maximum entropy [29, 30]. Ecologists are not always fortunate enough to have full presence-absence data for the species they study, and many data sets only have locations of species presence, referred to as presence-only data [31]. In an attempt to address this limitation, Royle and colleagues [31] introduced MaxLike, a formal likelihood model that explicitly estimates the probability of species occurrence and species’ prevalence, given presence-only data and a set of environmental covariates measured at each sampling location. They showed that MaxLike is capable of accurately estimating the absolute probability of occurrence (PO), i.e. the probability that a given species is present in a grid cell, whereas MaxEnt cannot [32]. Potential distribution maps of *C*. *odorata* were obtained using the packages “maxlike” ver. 0.1–5, “raster” ver. 2.3–12, “rgdal” ver. 0.9–1, “sp” ver. 1.0–16 and “tcltk2” ver. 1.2–10, in the software R ver. 3.1.2 [33].

We used presence records for *C*. *odorata* from the XAL Herbarium at the Institute of Ecology A.C. (INECOL) and from the Global Biodiversity Information Network (REMIB-CONABIO, Mexico) suitably geo-referenced and taxonomically determined by its herbarium specimen. We verified the coordinates of each record by visual inspection with a Geographic Information System to confirm its location on the terrain, obtaining 121 valid records. We used climate grids from the Moscow Forestry Sciences Laboratory (available URL: http://forest.moscowfsl.wsu.edu/) because this particular climate model was developed especially for Mexico and its periphery [34].

We used the three General Circulation Models (GCM) that are currently available for Mexico and three different scenarios of the Special Report on Emissions Scenarios (SRES) [35]. We used the Canadian Center for Climate Modeling and Analysis (CGCM3) version of scenarios A2 and B1. From the Geophysical Fluid Dynamics Laboratory (GFDLCM21), we used their version of the A2 scenario. Finally, from the Hadley Center (HADCM3) we used their version of scenarios B2 and A2. We explored projections for the years 2030 and 2060 for each of the indicated scenarios. All scenarios used, predict a future increase in average temperature, with HADCM3 A2 2060 showing the largest difference in comparison to current values, with an increase of 2.9°C. All models predicted a future reduction in precipitation. The scenarios of the HADCM3 model had the lowest decreases in precipitation, while those of CGCM3 had the largest, with almost two times less precipitation than the previous model. The scenario with the lowest future emission of CO_2_ is B1, and that with the highest is A2.

To select the climate variables for modeling we did a preliminary calibration with 200 iterations of the MaxLike algorithm [31], using the eleven variables available in the Moscow FSL dataset (see above) to describe current conditions and using the coordinates of verified records for *C*. *odorata*. We selected the models that converged, had no missing data and had less than 10% omission errors in their predictions. The calibration results showed that the most important variables were: degree-days> 5°C (hereafter: DD5), mean minimum temperature in the coldest month (MMIN), degree-days <0°C (MINDD0) and mean temperature in the warmest month (MTWM).

We generated models of potential distribution based on the four variables selected during calibration and z-standardized (i.e., mean = 0 and standard deviation = 1) all bioclimatic variables, current and future; i.e. for the projected value of each variable we subtracted the mean and then divided it by the standard deviation of the current data subset. Subsequently, in each of the 500 iterations of the final process we randomly selected 65% of the records for training and the remaining 35% for cross validation. The formula used to generate models of potential distribution was:

$$\begin{array}{l} \text{maxlike}(:\text{DD5}+\text{I}\left({\text{DD5}}^{\text{2}}\right)+\text{MMIN}+\text{I}\left({\text{MMIN}}^{\text{2}}\right)+\text{MINDD0}+\text{I}\left({\text{MINDD0}}^{\text{2}}\right)+\\ \text{MTWM}+\text{I}\left({\text{MTWM}}^{\text{2}}\right),\text{ MOD}\_\text{ACT},\text{ PTOS}\_\text{MOD},\text{ method}="\text{BFGS}",\\ \text{removeDuplicates}=\text{TRUE},\text{ savedata}=\text{TRUE}) \end{array}$$

Where “MOD_ACT” is a “raster stack” with all the variables under current conditions and “PTOS_MOD” is a “data frame” with the X and Y coordinates of the presence locations. For more details about the formula see the “maxlike” package.

If the resulting model fulfilled the following assumptions: a) convergence, b) had no missing data, c) omission value less than or equal to 10; then the model coefficients were used to project over the potential future instance of the species niche.

For example, this is the CGCM3 model under scenario A2 projected to 2030:

$$\begin{array}{l} \text{CGC}\_\text{A2}\_2030=\left(\text{INTER}\right)\text{+}\left(\text{COEF}\_\text{1aV}*\text{CA230}\_\text{DD5}\right)\text{+}\left(\text{COEF}\_\text{1aVC}*\left({\text{CA230}\_\text{DD5}}^{\text{2}}\right)\right)\text{+}\\ \left(\text{COEF}\_\text{2aV}*\text{CA230}\_\text{MMIN}\right)\text{+}\left(\text{COEF}\_\text{2aVC}*\left({\text{CA230}\_\text{MMIN}}^{\text{2}}\right)\right)\text{+}\\ \left(\text{COEF}\_\text{3aV}*\text{CA230}\_\text{MINDD0}\right)\text{+}\left(\text{COEF}\_\text{3aVC}*\left({\text{CA230}\_\text{MINDD0}}^{\text{2}}\right)\right)\text{+}\\ \left(\text{COEF}\_\text{4aV}*\text{CA230}\_\text{MTWM}\right)\text{+}\left(\text{COEF}\_\text{4aVC}*\left({\text{CA230}\_\text{MTWM}}^{\text{2}}\right)\right) \end{array}$$

INTER, which is the intercept, COEF_1aV is the coefficient of the first variable, COEF_1aVC is the coefficient of the first squared variable, and so on.

Finally, to obtain the values for the probability of future occurrence we applied the inverse of the link function used in MaxLike, which is the log function, hence:

Occurrence_CGC_A2_2030=exp(CGC_A2_2030)/(1+exp(CGC_A2_2030))

We arranged the models that satisfied the selection criteria according to their relative occurrence area (ROA) values; which is the ratio between the area of occurrence and the whole study area [36]. Then we chose 10 models around the statistical median that had an average probability of presence obtained with validated records nearest to 1 since theoretically the average of this value should be 1. Then we produced a consensus map by averaging these 10 maps (the same models set for current and future conditions). Finally, we regarded an indication of the likely presence of *C*. *odorata* to be the minimum probability value that generated a distribution map that included all of the actual presence records of the species in our data set.

A technique for evaluating models based solely on appearances by an array of modified confusion matrix are ROC curves (Receiver Operating Characteristic) [37]. This technique, which originated in radar signal processing, has been successfully applied in evaluating distribution models based on presence-absence algorithms [38, 39, 40], as well as presence-only data [29]. However, due to some problems with ROC analyses [41] we used partial-area ROC [42] to evaluate the current model by randomly selecting 35% of the records used in model generation and the current presence model. Partial-ROC is a modification of the original ROC curve that has been proposed to overcome two of the problems detected in the latter to evaluate SDMs [36], namely the inclusion in the calculation of the AUC of the full spectrum of proportional areas in the study area and an equal weighting of the omission and commission error components (see [41] for further details).

We superimposed the presence consensus maps for current and future conditions over official cartography of land use and vegetation cover [43] to identify those areas without any forests or tree cover that we regarded as prime candidates for reforestation or suitable for *C*. *odorata* plantations. Finally, we explored suitability for plantations by converting potential current areas into polygons, from which we subtracted the polygons located within protected areas under federal jurisdiction [44], as well as plots with slopes too steep for *C*. *odorata* and those that were too far from roads (which would make plantation maintenance and exploitation too costly). Thus, we chose polygons with a PO greater than 0.85, slopes less than 10° and that were no further than 10 km from any main road. To validate our potential distribution model with external data and to explore the potential impact of climate change with current policies, the model was contrasted with the polygons of *C*. *odorata* plantations officially registered from 2000 to 2014, based on information provided by CONAFOR. These plantations were established by the owners without any technical advice from CONAFOR. They chose plots to introduce red cedar based on the knowledge they had acquired about the habits of the species from experience.

Finally, to analyze the direction and magnitude of change between the maps of current and future potential distributions, we calculated and compared the centroids and vectors of current and future suitable areas using a python-based GIS toolkit, SDMtoolbox [45]. This analysis is used to summarize the core distributional shifts in the range of many species. It associates each species distribution pattern with a centroid (expected multivariate location under the model fitted) and creates a vector file depicting the magnitude and direction of the predicted change over time as a function of scenario values.

## Results

To produce the best fitting niche model possible we conducted cross validations to assess the quality of the models. We used 65% of the sample of *Cedrela*’s true locations for training and the remaining 35% for testing (i.e. validation). The lowest omission error of the best 10 models for current climate conditions was 2.32, the largest was 9.3, and the average was 5.11. The ROC partial evaluation returned a value of *p* <0.002. The current conditions model produced a minimum value of 0.51 for the PO of *C*. *odorata*. Therefore, we regarded this value as indicative of the "likely presence" of *C*. *odorata* both in the current map and those projected for future conditions. This way, we estimated the potential distribution area of the species, which was 573,079 km^2^ under current conditions, and ranged from 551,053 km^2^ to 650,356 km^2^ for the different scenarios of future climate change (Table 1).

**Table 1:** Potential distribution area, average (±sd) probability of occurrence and average elevation (±sd) of *C*. *odorata* distribution throughout Mexico under different climate scenarios.

Model and scenario	Area (km^2^)	% difference in area between current and future model	Average probability of occurrence	Average elevation (m a.s.l.)
**Current**	573,079	----	0.93 (±0.13)	462 (±584)
**CGCM3 B1 2030**	551,053	- 3.84	0.88 (±0.15)	424 (±538)
**CGCM3 B1 2060**	583,601	+ 1.84	0.85 (±0.15)	510 (±631)
**CGCM3 A2 2030**	605,370	+ 5.63	0.88 (±0.15)	508 (±627)
**CGCM3 A2 2060**	585,565	+ 2.18	0.79 (±0.15)	540 (±646)
**GFDLCM21 A2 2030**	644,573	+ 12.48	0.90 (±0.14)	593 (±700)
**GFDLCM21 A2 2060**	593,782	+ 3.61	0.83 (±0.14)	548 (±661)
**HADCM3 B2 2030**	588,817	+ 2.75	0.87 (±0.15)	451 (±548)
**HADCM3 B2 2060**	650,356	+ 13.48	0.86 (±0.14)	546 (±627)
**HADCM3 A2 2030**	606,007	+ 5.75	0.87 (±0.14)	510 (±615)
**HADCM3 A2 2060**	643,720	+ 12.33	0.77 (±0.15)	542 (±621)

**Fig 2:**
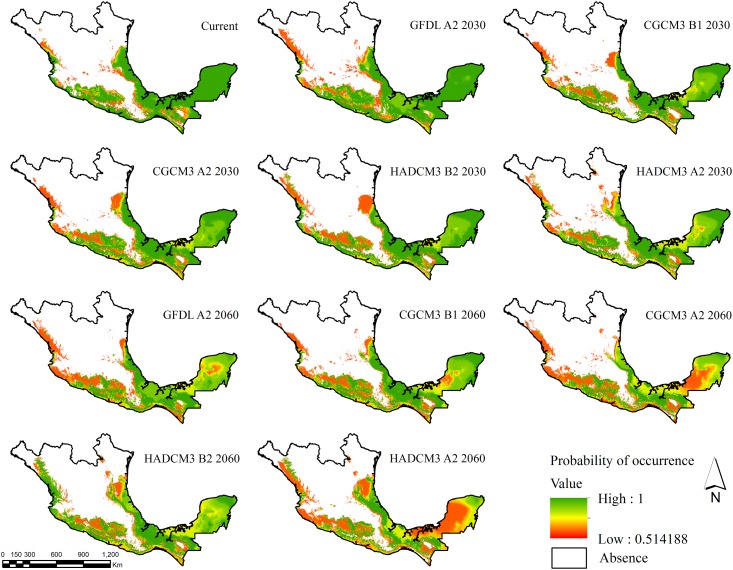
Map of the potential distribution of *C*. *odorata* in Mexico under different global change scenarios projected for 2030 and 2060.

All of the scenarios of future climate change that we tried suggested that *C*. *odorata* will expand across Mexico, with the exception of CGCM3 B1 by 2030. Under the latter scenario, the expected area decreased by 3.8% in relation to the estimated current distribution. The models with the largest projected area were HADCM3 A2 and HADCM3 B2 by 2060 with an increase of 12.3% and 13.5%, respectively (Table 1 and Fig 2). The average probability of occurrence for each of the scenarios decreased over time. The smallest probability, 0.77 (± 0.15), was associated with HADCM3 A2 by 2060. Most models showed a future increase in the average elevation of *Cedrela*’s occurrence in relation to the projected current elevation, the only exceptions were CGCM3 B1 and HADCM3 B2, both projected a decrease in elevation by 2030. We also found that we could expect a further increase by 2060 in the average elevation our models projected for 2030, except in the case of GFDLCM21 A2.

**Fig 3:**
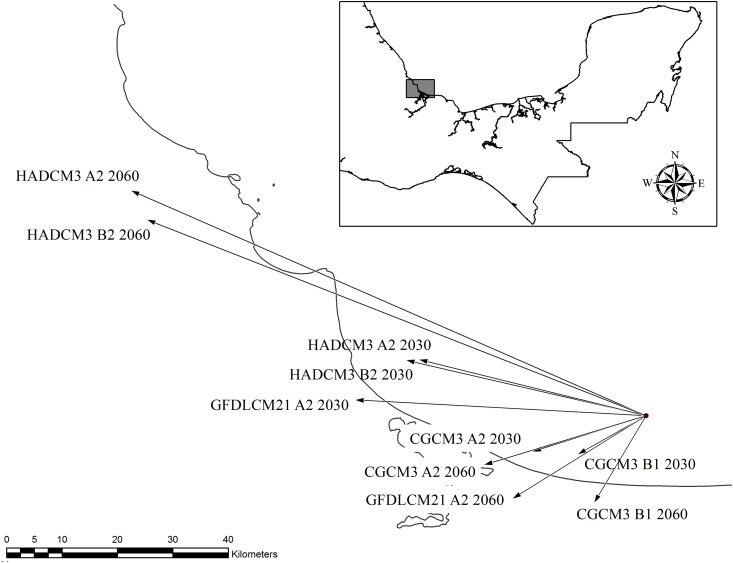
Centroid location, and vector direction and magnitude for each potential distribution map of *C*. *odorata* in Mexico. Projection models and scenarios are abbreviated (see text) and model years are given.

The centroid of the current distribution map of *Cedrela* was located at 18°49'30'' N latitude and 95°27'50.40'' W longitude, 12 km from the coast in the Gulf of Mexico. The direction of the vectors obtained with the SDMTools contrasted for each of the GCM selected in this study. The vectors of the CGCM3 model pointed to the southwest, while those of HADCM3 pointed to the northwest. The GFDLCM21 vectors differed among years: that for 2030 pointing to the west while that of 2060 shifted towards the southwest. Regarding vector magnitude, the largest intensity of change occurred in the vectors related to the A2 and B2 scenarios projected to 2060 under HADCM3 (Fig 3).

*C*. *odorata* grows in different vegetation types, so when we superimposed our current distribution map of *Cedrela* over INEGI’s Series V (2013) land use and vegetation cover map [43], we found some polygons with woody vegetation, some with secondary vegetation and others with no apparent vegetation. Table 2 shows only the sites where woodland or forest cover is not dominant, which we regarded as suitable for the establishment of *C*. *odorata* plantations. The INEGI Series V [43] categories: “selva alta” and “selva mediana” for the evergreen (“perennifolia”) and semi-evergreen (“sub-perennifolia”) vegetation types were all regarded here as tropical evergreen forest.

**Table 2:** Land cover and vegetation types with a high potential for the establishment of *C*. *odorata* plantations in Mexico.

Land cover and vegetation types	Area (km^2^)					
	Current	CGCM3		GFDLCM21 A2 2060	HADCM3	
		B1 2060	A2 2060		B2 2060	A2 2060
**Shrubby secondary vegetation, derived from:**						
Tropical evergreen forest	14,581	14,327	14,119	13,346	14,888	14,877
Tropical deciduous forest	33,746	35,373	35,902	35,189	38,288	38,094
**Subtotal**	**48,327**	**49,701**	**50,022**	**48,535**	**53,176**	**52,971**
**Herbaceous secondary vegetation, derived from:**						
Tropical evergreen forest	644	617	605	517	671	679
Tropical deciduous forest	198	199	206	208	252	244
No apparent vegetation	976	983	899	997	1,017	851
**Subtotal**	**1,819**	**1,801**	**1,712**	**1,724**	**1,940**	**1,775**
**Total**	**50,146**	**51,502**	**51,734**	**50,260**	**55,117**	**54,746**

The largest contributions in area for sites that we regarded as optimal for the establishment of *Cedrela* plantations, were made by secondary shrubby vegetation growing in old-fields that were established in areas originally covered by tropical evergreen or tropical deciduous forest, and had usually been abandoned for less than 20 years. Altogether, this area accounts for 48,237 km^2^ in our current conditions projection, and increased to 53,176 km^2^ by 2060 in the HADCM3 B2 model. The secondary shrubby vegetation derived from tropical deciduous forest was the only category for which there was a consistent increase in the potentially usable area in all future projections with respect to the current conditions (Table 2). Finally, by excluding the polygons of Natural Protected Areas under federal jurisdiction and sites that were unsuitable due to their steep slopes and isolation from main roads, we delimited 4,885 suitable polygons that span an area of 35,377 km^2^.

In Mexico, from 2000 to 2015, the *C*. *odorata* forest plantations that were recognized and financially supported by CONAFOR covered an area of 200.9 km^2^ in 6,271 different polygons. After superimposing these on the current potential distribution map, we found that the potential distribution of *C*. *odorata* from this study has a very good match with 95% of them (5,993 polygons) spanning an area of 195.3 km^2^.

## Discussion

### Potential future distribution

Most of the *Cedrela odorata* models tested showed an increase in the potential distribution area of the species by 2030 and 2060. This projected increase in area is related to an expected increase in warm-humid areas or represents an upward vertical shift into what is today covered by temperate vegetation [20, 21]. Similarly we found a likely increase in the presence of red cedar in areas that today correspond to dry or semi dry warm areas.

The largest increases in area for the 2060 scenarios account for between 12.3% and 13.5% of *C*. *odorata*’s current distribution when using the HADCM3 general circulation model for A2 and B2 climate scenarios, respectively. The HADCM3 future model projects large increases in average temperature but only a slight reduction in precipitation, so under these future conditions *C*. *odorata* should find better conditions. However, the average value for the future probability of occurrence is lower in all of our models. Most of them, however, suggest an increase in elevation is likely [46]. This increase in the elevation range of *Cedrela* predicted by most of the scenarios we used is consistent with the findings of Gómez and colleagues [47]. They found an increase in the elevation of this species over areas on the eastern slopes of the Sierra Madre Oriental, where conditions are favorable for *C*. *odorata*.

### Mitigation potential

The assessment of the probability of occurrence of *C*. *odorata* with MaxLike suggests that this species has significant potential to help mitigate the effects of climate change. Its adaptability, fast growth, valuable wood, and initially branchless habit suggest it is well suited for timber plantation [48]. It is a heliophytic tree that is regarded as a persistent pioneer due to its relatively long life span. Its establishment and growth are favored in medium to large canopy gaps after natural disturbances, and in agricultural landscapes it can also thrive if proper agroforestry practices are followed. Since pre-Hispanic times its wood has been prized and in traditional shifting cultivation farmers usually spare *C*. *odorata* saplings, juveniles and adults during forest felling [49], explaining its relatively high density in man-made landscapes. A significant portion of Mexico’s territory is suitable for increasing carbon sequestration with *C*. *odorata* plantations. These plantations could be monospecific or ideally multi-specific, combining species that have similar requirements.

Our potential distribution models for future scenarios also reveal there is 50,000 to more than 55,000 km^2^ of land in the country that is currently without any tree cover and where the potential for successfully growing *Cedrela* trees is high. The areas we identified have the additional potential to increase the country's forested area, reduce soil erosion, and generate economic revenue and goods, while helping in the conservation of biodiversity [13]; thus, supporting both the mitigation of and adaptation to climate change in those areas. Climate change mitigation is very important, as Warren and colleagues [50] showed that without mitigation 57±6% of plants and 34±7% of animals in their study are likely to lose more than 50% of their present climatic range by the 2080s. However, they also found that by applying mitigation measures losses can be reduced by 60%.

Over an eight year period, *C*. *odorata* can store 4.45 tons of C ha^-1^ [51]. Thus, we estimate that in the 35,377 km^2^ where we found have optimum conditions for its establishment, it could store approximately 15,743,094 tons of carbon over the same time span. Regarding mitigation activities, Xu and colleagues [11] found that the activity with the least investment required per ton of C is forest regeneration, followed by a long-rotation plantation cycle and forest protection. Given this, we suggest that *C*. *odorata* plantations would increase carbon capture with a potentially competitive investment cost per ton of carbon captured.

The polygons we identified with the potential to grow *C*. *odorata* coincide with the known requirements of this species as a light demanding tree that can be planted in open areas [52]. However, we did not take soil type into account in this study, nor did we address the probability of flooding, which *C*. *odorata* cannot tolerate. Rather than promoting monocultures of this species, we are suggesting the use of a mix of tree species in plantations. This practice has been documented to reduce the risk of borer attack (*Hypsipyla grandella* Zeller, Lepidoptera Phycitidae), mainly during the first two to three years of tree growth after planting [52].

The behavior of the MaxLike algorithm has been tested by Fitzpatrick and colleagues [53]. They found, at least for ants in New England, a more sensible prediction of species distribution when using MaxLike in comparison with other algorithms. In our case, when we overlapped *C*. *odorata* plantation locations with our expected distribution maps the match was greater than 95%. We interpret this result to mean that MaxLike succeeded very well at matching the knowledge of the people in the region regarding the distribution patterns of this species.

MaxLike should be further tested, as has been done with other algorithms, in order to assess its performance in ecological niche modeling. We think it is an interesting addition to the wide spectrum of tools available for projecting the distribution of species into future scenarios of climate change. For species distribution modeling, MaxLike and other models that are based on an explicit sampling process [54, 55] should be considered important alternatives to the widely used MaxEnt framework [53].

## Conclusions

Our modeling efforts suggest that it is very likely that by 2060 *C*. *odorata* will still find suitable climate conditions in Mexico, and this may allow for its increased presence in the landscape. This increase is very likely to imply not only a larger spatial distribution but also its presence at higher elevations in mountain ranges. *C*. *odorata* is a tree species of ecological, economic, and social relevance, and therefore it is a valuable target to include in strategies to mitigate climate change at the regional and national levels. The use of the MaxLike algorithm in species distribution modeling was an excellent choice for the analysis of the response of this species to climate change scenarios in our study, but it may still need to be evaluated under a variety of conditions, as this has been necessary for other algorithms.

## Supporting Information

S1 File *Cedrela odorata* occurrence data. (CSV)

S2 File Potential distribution models with MaxLike. (R)
